# Photosynthetic Temperature Tolerance Threshold Determines How Isoprene Emission is Affected by Elevated CO_2_ Concentration at High Temperatures

**DOI:** 10.1002/pei3.70053

**Published:** 2025-05-02

**Authors:** Vinícius Fernandes de Souza, José Francisco de Carvalho Gonçalves, Bakhtier Rasulov, Eero Talts, Catherine Morfopoulos, Sérgio Duvoisin Junior, Patrícia Melchionna Albuquerque, Ülo Niinemets

**Affiliations:** ^1^ Institute of Agricultural and Environmental Sciences Estonian University of Life Sciences Tartu Estonia; ^2^ Laboratory of Plant Physiology and Biochemistry National Institute for Amazonian Research – INPA Manaus Brazil; ^3^ Imperial College of London, Department of Life Sciences (Silwood Park) Berks UK; ^4^ Amazonas State University Manaus Brazil; ^5^ Estonian Academy of Sciences Tallinn Estonia

**Keywords:** BVOCs, dimethylallyl diphosphate, energy flux, isoprene synthase, post‐illumination, tropical and temperate species

## Abstract

The suppression of isoprene emissions by high CO_2_ levels can be mitigated by increasing temperature; however, little is known about why and to what extent species differ in their temperature‐dependent release from high CO_2_ inhibition. We studied leaf photosynthetic characteristics and isoprene emissions over a 25°C–40°C temperature range at CO_2_ concentrations of 150, 400, and 1000 μmol mol^−1^ in two species with contrasting heat resistance. In the temperate species *Populus tremula* , rising temperatures above 30°C shifted electron flow from photosynthesis to isoprene synthesis, reducing CO_2_ inhibition due to enhanced isoprene synthase activity and decreased sensitivity of the DMADP pool. Conversely, the tropical species *Inga edulis* showed greater heat tolerance in its photosynthetic apparatus, maintaining electron flow for CO_2_ fixation, and exhibited a consistent CO_2_ suppression of isoprene emissions throughout the experiment. These findings indicate that species differences in relative sensitivity of light and dark reactions of photosynthesis play crucial roles in modulating isoprene emissions under combined high CO_2_ and temperature conditions.

## 1 Introduction

The short‐term effects of temperature on leaf isoprene emissions are well documented, but CO_2_‐driven changes remain less understood. Typically, isoprene emissions decrease with elevated CO_2_ and increase with rising temperatures, suggesting that future warming may offset CO_2_‐induced suppression. However, variability in responses across species and limited mechanistic understanding hinder accurate predictions. Although this variation is not fully understood, the CO_2_ sensitivity of isoprene appears to be largely dependent on the dimethylallyl diphosphate (DMADP) pool size that each species can support (Niinemets et al. 2021). DMADP, synthesized through the chloroplastic methylerythritol 4‐phosphate (MEP) pathway, serves as the immediate precursor for isoprene formation via isoprene synthase (IspS) (Perez‐Gil et al. 2024).

Temperature primarily affects isoprene emission through the availability of DMADP and the activity of IspS (Rasulov et al. 2010; Li et al. 2011), while elevated CO_2_ typically reduces DMADP pool size (Rasulov et al. 2009; de Souza et al. 2018; Niinemets et al. 2021), sometimes accompanied by an accumulation of upstream MEP pathway intermediates such as hydroxymethylbutenyl diphosphate (HMBDP) (Sahu et al. 2023). Earlier hypotheses pointed to substrate limitations, such as pyruvate availability (Rosenstiel et al. 2003; Wilkinson et al. 2009; de Souza et al. 2018) or restricted Pi recycling (Li and Sharkey 2013a; Morfopoulos et al. 2013; Monson et al. 2016; Rasulov et al. 2018)—but these mechanisms alone do not fully explain the short‐term suppression of isoprene under elevated CO_2_, particularly at high temperatures. Instead, chloroplastic energetic imbalance and temperature‐dependent IspS regulation have emerged as central factors shaping isoprene responses under these conditions (Niinemets et al. 1999b; Selmar and Kleinwächter 2013; Dani et al. 2014, 2015).

While such imbalance is sometimes associated with reduced PSII activity and lower DMADP levels (Rasulov et al. 2009), recent findings show that isoprene suppression can also occur independently of electron transport limitations (Lantz et al. 2019; Sahu et al. 2023). Yet, even in the absence of photochemical constraints, elevated CO_2_ can intensify carbon assimilation demands, thereby increasing ATP and NADPH consumption, which reduces their availability for MEP flux and ultimately limits DMADP synthesis (Harrison et al. 2013).

Importantly, the degree of isoprene inhibition at high CO_2_ varies among species and can be influenced by growth temperature. Plants grown in warmer conditions tend to exhibit greater CO_2_ sensitivity than those grown in cooler environments (Staudt et al. 2022). Some species show decreased sensitivity at moderate temperatures (25°C–30°C), while others maintain suppression at higher temperatures (35°C–40°C) (Rasulov et al. 2010; Way et al. 2011; Sun et al. 2013; Potosnak et al. 2014; Monson et al. 2016). This interspecific variation has been consistently associated with differences in species' thermal tolerance to a given increase in temperature (Potosnak 2014). However, the physiological basis for this response remains uncertain, and the mechanisms regulating the interplay between CO_2_ suppression and temperature are still debated (Way et al. 2011; Potosnak 2014; Sharkey and Monson 2014; Lantz et al. 2019; Sahu et al. 2023).

One consistent observation across studies is that the reduction in CO_2_ sensitivity becomes more pronounced as temperatures exceed the photosynthetic optimum, often in interaction with additional environmental constraints such as high light and drought (Sun et al. 2012; Dani et al. 2014). Although high CO_2_ alleviates some limitations in photosynthesis, temperatures beyond the plant's tolerance can still impose energetic pressure, altering the balance between photochemical energy generation and carbon assimilation (Lewis et al. 2015; Staudt et al. 2022; de Souza et al. 2024). For instance, in black poplar under heat stress, elevated CO_2_ suppressed photosynthesis while isoprene emissions remained high (Portillo‐Estrada 2024). This apparent decoupling, where isoprene emission persists despite photosynthetic suppression, has been partly attributed to the use of alternative carbon sources such as cytosolic DMADP import (Rosenstiel et al. 2003; Brilli et al. 2007; Trowbridge et al. 2012; de Souza et al. 2018; Portillo‐Estrada 2024). However, such contributions are generally minor and likely relevant only under extreme or prolonged stress (Brilli et al. 2007; Yáñez‐Serrano et al. 2019), making them insufficient to explain sustained peak emission rates. Given the small carbon requirement for isoprene synthesis, the short‐term loss of CO_2_ suppression above the thermal tolerance threshold is more likely driven by a shift in energy use, whereby reduced carboxylation frees excess reducing power that is redirected toward the MEP pathway.

Similarly, drought‐sensitive species exhibit increased isoprene emissions as carbon assimilation declines, reflecting a shift in electron flow toward alternative sinks (Dani et al. 2014). These findings suggest a regulatory mechanism in which the loss of suppression likely stems from shifts in energy allocation dynamics, where overflow from excess reducing power is increasingly diverted to alternative sinks. As isoprene synthesis requires relatively little ATP and NADPH compared to carbon assimilation, it often relies on excess reducing power. As temperatures surpass the photosynthetic optimum, reduced carbon assimilation frees up reducing power, which is redirected toward DMADP synthesis and consequently stimulates isoprene emission, weakening CO_2_ suppression (Niinemets et al. 1999a; Selmar and Kleinwächter 2013; Dani et al. 2015). This relationship, driven by energetic imbalances resulting from high light, low CO_2_, drought, and heat stress, underscores the role of excess reducing power in elevated isoprene output (Sharkey and Loreto 1993; Rasulov et al. 2009; Sun et al. 2012; Harrison et al. 2013).

Moreover, enzymatic regulation—including temperature‐dependent increases in IspS activity—may further compensate for substrate limitations (Rasulov et al. 2010; Li et al. 2011). Thus, understanding isoprene responses under combined CO_2_ and thermal stress requires integrating both energetic balance and key enzymatic controls within the MEP pathway.

Although several studies have linked isoprene emission to chloroplast energy status, few have quantitatively assessed how electron partitioning affects CO_2_ suppression of isoprene emissions at higher temperatures. This study focuses on the mechanisms underlying the loss of CO_2_‐induced isoprene suppression at higher temperatures, emphasizing both energetic and enzymatic controls. To test this, we selected the tropical Amazonian *Inga edulis* and the temperate European *Populus tremula* . We aimed to assess (a) the responsiveness of photosynthetic traits and isoprene emission rates to elevated CO_2_ and temperature, (b) the combined effects on energy partitioning between carboxylation, DMADP pool size, and isoprene production, and (c) the temperature dependence of isoprene synthase activity. Comparing these responses provides insights into species‐specific mechanisms, forming a basis for future studies to evaluate whether similar patterns occur in other taxa and conditions.

## 2 Materials and Methods

### 2.1 Plant Material and Experimental Setup

The experiments were conducted using two contrasting broad‐leaved tree species: the tropical species *Inga edulis* Mart. and the cool‐temperate species *Populus tremula* L. Inga edulis is a fast‐growing, evergreen pioneer species known for its ability to fix substantial amounts of nitrogen (N_2_), and it is widely utilized for its fruits, wood, shade, and as a component in agroforestry systems (Lojka et al. 2010). This species is prevalent in the Amazon Basin and the Guiana Shield, where it plays a significant role in the ecological dynamics and biodiversity of the rainforest (Lojka et al. 2010). As one of the most species‐rich genera and the largest in terms of tree individual numbers in the Amazon region, *Inga* is crucial for understanding tropical forest ecology (Steege et al. 2013; Cardoso et al. 2017). The species' ability to thrive in nutrient‐poor soils and its high efficiency in utilizing light and CO_2_ make it an ideal candidate for studying isoprene emission responses to elevated CO_2_ and temperature conditions (Garcia et al. 2019).

*Populus tremula* , a deciduous tree species, is distributed widely across temperate and boreal regions and is found in both natural forests and plantations (Worrell 1995). Poplar species, including *P. tremula*, are recognized as a primary model system in plant science, particularly for studies in tree physiology and responses to environmental stressors, such as elevated CO_2_ and temperature (Niinemets and Sun 2015; Lauriks et al. 2021; de Souza et al. 2018). Poplars' broad geographical distribution and adaptability make them an excellent model for studying the physiological controls on isoprene emissions under varying environmental conditions.

For both species, their contrasting ecological adaptations— *I. edulis* thriving in tropical, high‐temperature environments with high light availability and *P. tremula* adapted to temperate climates with more variable temperatures—provide a comprehensive framework for examining species‐specific responses in isoprene emission. The differing thermal tolerances and physiological strategies of these species are expected to offer insights into how isoprene emissions may shift under future climate scenarios as characterized by increased CO_2_ levels and temperature fluctuations (de Souza et al. 2024).

Measurements in *Inga edulis* trees were performed at the National Institute for Amazon Research (INPA) in Manaus, Brazil (3°8′ S, 60°0′ W). During the study period in the dry season, daily temperatures ranged from 26°C to 36°C, with an average of approximately 34°C. Solar radiation was consistently high, frequently exceeding 1500 μmol m^−2^ s^−1^, and relative humidity averaged around 70%. Monthly precipitation was below 100 mm, reflecting the warm and sunny conditions typical of the dry season in the Central Amazon.

Experiments with *Populus tremula* trees were conducted at the Estonian University of Life Sciences in Tartu, Estonia (58.3833° N, 26.7167° E). During the study period in September, daily temperatures ranged from 5°C to 22°C, with an average of approximately 13°C. Solar radiation peaked at around 1400 μmol m^−2^ s^−1^, and relative humidity averaged 83%. Monthly precipitation was approximately 58 mm, reflecting the mild and moderately humid conditions of the region in early autumn.

Trees of both *I. edulis* and *P. tremula* were grown outdoors under full sun conditions. For the experiments, small branches were detached in the morning, immediately re‐cut under water to prevent embolism formation, and transported to the laboratory in water‐filled containers. Upon arrival, branches were re‐cut underwater to restore xylem continuity and minimize potential disruptions to water transport. Branches were selected from the upper third of the canopy, exposed to full sunlight, and only fully expanded, physiologically mature leaves with no signs of senescence, herbivory, or mechanical damage were used. Measurements were conducted between 09:00 and 17:00 h. Given the prompt handling and hydration protocol, leaves remained physiologically active during measurements, as evidenced by stable gas exchange and fluorescence parameters. For both species, one leaf was measured from each of three to five individual trees.

### 2.2 Leaf Gas Exchange Analyses and Isoprene Emission Rates

For *I. edulis* , leaf gas exchange and isoprene emission rates were measured using a LI‐6400XT portable open gas exchange system with a LI‐6400‐40 leaf chamber fluorometer that has 2 cm^2^ chamber windows (LI‐Cor Inc., USA), coupled with a Proton‐Transfer‐Reaction Quadrupole Mass Spectrometer (PTR‐QMS, Ionicon Analytik, Austria). The system flow rate was set to 400 μmol s^−1^, and the leaves were adapted to a photosynthetic quantum flux density (*Q*) of 1000 μmol m^−2^ s^−1^ (10% blue light). The PTR‐QMS was connected to the LI‐6400XT exhaust via a Teflon T‐piece, following the configuration and calibration protocol of Jardine et al. (2014). In brief, the PTR‐QMS was operated with a drift tube voltage of 600 V, a temperature of 40°C, and a pressure of 2.0 mbar. Isoprene (m/z 69) was monitored with a dwell time of 2 s, and the instrument was calibrated using a certified 1 ppmv isoprene standard (Restek) diluted in humidified zero air to six concentrations ranging from 0 to 10.5 ppbv. Fast temperature changes in the leaf chamber were achieved by using temperature‐controlled water jackets attached to Peltier cooler blocks. Background isoprene levels were measured before and after each leaf measurement under the same conditions, and subtracted from leaf measurements. CO_2_ diffusion leaks in the gaskets of the LI‐6400XT leaf chamber were corrected according to Bellasio et al. (2016), which involved adjusting the net assimilation rate and recalculating the intercellular CO_2_ concentration (*C*_i_).

For *P. tremula* , measurements were conducted with a custom two‐channel gas‐exchange system (Laisk et al. 2002) combined with PTR‐QMS (Ionicon Analytik, Austria). Leaves were enclosed in an 8.04 cm^2^ cuvette with the upper leaf surface in contact with a starch gel, allowing for optimal heat exchange. The leaf chamber water jacket temperature was controlled by two circulating water baths, allowing for rapid changes in leaf temperature on the order of a few seconds. Gas flow was maintained at 500 μmol s^−1^. CO_2_ exchange was monitored using a LiCor LI‐6252 analyzer, and the transpiration rate was measured with a custom‐made psychrometer. PTR‐QMS settings allowed a time resolution of 1.3 s and a detection limit of approximately 10 pmol mol^−1^. Calibration was performed using a standard gas containing 3.43 ppm isoprene in N_2_. With two identical gas channels (measure and reference), the background isoprene concentrations were frequently measured during each set of measurements.

In both systems, baseline conditions in the leaf cuvette were set to 21% O_2_, 400 μmol mol^−1^ CO_2_, ~60% relative humidity, 30°C leaf temperature, and 1000 μmol m^−2^ s^−1^ *Q*. Leaves were acclimated under these conditions for 20–30 min until gas exchange characteristics stabilized before measuring responses to changes in environmental factors.

### 2.3 Fluorescence Measurements

Chlorophyll fluorescence was measured simultaneously with gas exchange and isoprene emissions. For *I. edulis* , we used the integrated leaf chamber fluorometer of the LI‐6400XT system employing modulated light and saturating pulses (~8000 μmol m^−2^ s^−1^, 0.5 s duration) to determine the steady‐state fluorescence yield (*F*_t_) and maximum fluorescence in the light‐adapted state (*F*_m_′). For *P. tremula* , measurements were made with a Walz PAM‐101 fluorometer. Saturating pulses of white light (10,000 μmol m^−2^ s^−1^, 1 s duration) were applied to determine *F*_m_′, and *F*_t_ was recorded before each pulse.

For both species, the effective quantum yield of PSII (Φ_PSII_) was calculated as Φ_PSII_ = (*F*_m_′ − *F*_t_)/*F*_m_′, and electron transport rate (*ETR*) was determined as *ETR* = Φ_PSII_ × α × β × *Q* (Genty et al. 1989). In *ETR* calculations, leaf absorptance, α, was set at 0.85, and partitioning of absorbed quanta between the two photosystems, β, was set at 0.5.

### 2.4 Monitoring Photosynthesis and Isoprene Emission Under Different Temperatures and CO_2_ Concentrations

Measurements commenced by placing the leaf in the cuvette and establishing baseline conditions: *Q* of 1000 μmol m^−2^ s^−1^, leaf temperature of 30°C, ambient CO_2_ concentration (*C*_a_) of 400 μmol mol^−1^, oxygen concentration of 21%, and vapor pressure deficit (*VPD*) of approximately 1.7 kPa. The leaf was acclimated under these conditions until steady‐state values for net assimilation rate (*A*), stomatal conductance (*g*_s_), *C*_i_, and isoprene emission rate, *I*, were reached, typically within 20–30 min. The steady‐state values were recorded, and temperature response curves were measured by changing leaf temperature in a sequence of 25°C, 30°C, 35°C, and 40°C under each CO_2_ concentration (150, 400, and 1000 μmol mol^−1^). At each temperature step, measurements were recorded once steady‐state conditions were reestablished, typically requiring approximately 8 min per temperature point. This approach ensured that physiological responses to temperature and CO_2_ variations were accurately captured for every leaf, with sufficient time allowed for stabilization at each experimental condition.

To estimate the maximum carboxylation rate (*V*_cmax_), we applied the “one‐point method” by De Kauwe et al. (2016), adjusting for thermal sensitivity using average temperature parameters based on the habitat origins of *P. tremula* and *I. edulis* (Galmés et al. 2016). These temperature‐specific adaptations enable the precise incorporation of Rubisco's thermal response, thereby improving model predictions of photosynthetic performance under varying environmental conditions. With *V*_cmax_ determined, we calculated the electron transport rate (*J*_v_) following Morfopoulos et al. (2014), facilitating accurate partitioning of electron flux toward carboxylation. Specifically, we used the following equation:

(1) $$J_{v}=4V_{\text{cmax}}\left(\frac{C_{i}+2\Gamma^{*}}{C_{i}+K_{\text{m}}}\right)$$

where *K*_m_ and Γ* vary with leaf temperature, calculated as per Galmés et al. (2016). Finally, by determining *J*_v_, we isolated the non‐carboxylation electron flux as the difference between *ETR* and *J*_v_.

### 2.5 Dimethylallyl Diphosphate Pool Size and Isoprene Synthase Activity

Estimates of in vivo DMADP pool size and isoprene synthase (IspS) activity during temperature response curves at different CO_2_ concentrations were obtained using the methods reported in detail in Rasulov et al. (2010, 2011) and Weraduwage et al. (2022). The method for DMADP pool size estimation involves measurements of post‐illumination isoprene release after recording the steady‐state value of isoprene emission in light. The method is based on the assumption that the rapid initial dark decay of isoprene emission, for 250 s after leaf darkening, relies on the DMADP pool that has accumulated during the previous light period, and no new chloroplastic DMADP is formed in the dark during this initial period. Thus, the DMADP pool size supporting the isoprene emission rate in light was estimated as the integral of post‐illumination isoprene emission from the time of leaf darkening until approximately 250 s, when the initial isoprene dark release rate approached zero. The postillumination method is advantageous because it is non‐destructive, allowing for the measurement of DMADP pool sizes and isoprene synthase activities in the same leaves under varying physiological conditions.

The isoprene synthase rate constant (s^−1^) was estimated according to Rasulov et al. (2010). Paired values of isoprene emission and DMADP pool size remaining at this moment in time were obtained through the dark decay of isoprene release, and the rate constant was calculated from the slope of the linear regression between isoprene emission rate and DMADP pool size.

### 2.6 Data Analyses

To account for plant‐to‐plant variability and facilitate comparisons among treatments, all physiological variables were adjusted by dividing each value by the overall mean of each treatment, following the approach suggested by Loftus and Masson (1994). This normalization allows for a more precise comparison of treatment impacts by minimizing the influence of individual plant variation.

To investigate the temperature responses of key photosynthetic traits, including net assimilation rate, electron transport rate, and isoprene emission characteristics, an Arrhenius‐type response curve with an optimum was fitted as follows:

(2) $$y_{i}=\frac{e^{\left(c-\Delta H_{\text{a}}/\mathrm{RT}\right)}}{1+e^{\left(\mathrm{\Delta ST}-\Delta H_{\text{d}}\right)/\mathrm{RT}}}$$

where *y*_i_ is the dependent variable, *c* is the scaling constant, Δ*H*_a_ (J mol^−1^) is the activation energy, Δ*H*_d_ (J mol^−1^) is the deactivation energy, Δ*S* (J mol^−1^ K^−1^) is the entropy term, *T* (K) is the leaf temperature, *R* (8.314 J mol^−1^ K^−1^) is the gas constant. The optimum temperature (*T*_opt_) for each variable was determined as described by Niinemets et al. (1999a).

(3) $$y_{i}=\frac{∆H_{\text{d}}}{∆S+\mathrm{Rln}\left(\frac{∆H_{\text{d}}}{∆H_{\text{a}}}-1\right)}$$

To assess the temperature responses of biological processes, we calculated the reaction rate increase from *T* to *T* + 10 as follows:

(4) $$Q_{10,i}=e^{\frac{\Delta H_{\text{a}}\left[1/T-1/\left(T+10\right)\right]}{R}}$$

The Arrhenius‐type models were fitted by minimizing the residual sum of squares using Microsoft Excel Solver, with initial parameter estimates based on prior studies (Niinemets et al. 1999a; Rasulov et al. 2014). Although model fitting involves interdependent parameters, this approach provided consistent estimates of Topt and curve shape across replicates.

Multiple linear regression was applied to simultaneously assess the main effects and interactions between temperature and CO_2_, while simple linear regressions were used to explore temperature sensitivity at each CO_2_ concentration. Relationships between isoprene emission, DMADP pool size, and IspS activity were analyzed using linear regression.

Correlation analyses examined the interactions between photosynthesis, isoprene emission, and DMADP pool size, as well as changes in energy allocation across temperature and CO_2_ treatments. To compare treatment effects, paired‐sample t‐tests assessed the impacts of elevated CO_2_ at different temperatures within species, while independent sample t‐tests compared responses between species.

Means ± standard error are reported based on biological replicates (*n* = 3–5 plants). All analyses were conducted using SPSS software version 21.0 (SPSS Inc., Chicago, IL, USA), with statistical significance set at *p* < 0.05.

## 3 Results

### 3.1 Temperature and CO_2_ ‐Driven Variations in Photosynthesis and Electron Flux

**FIGURE 1:**
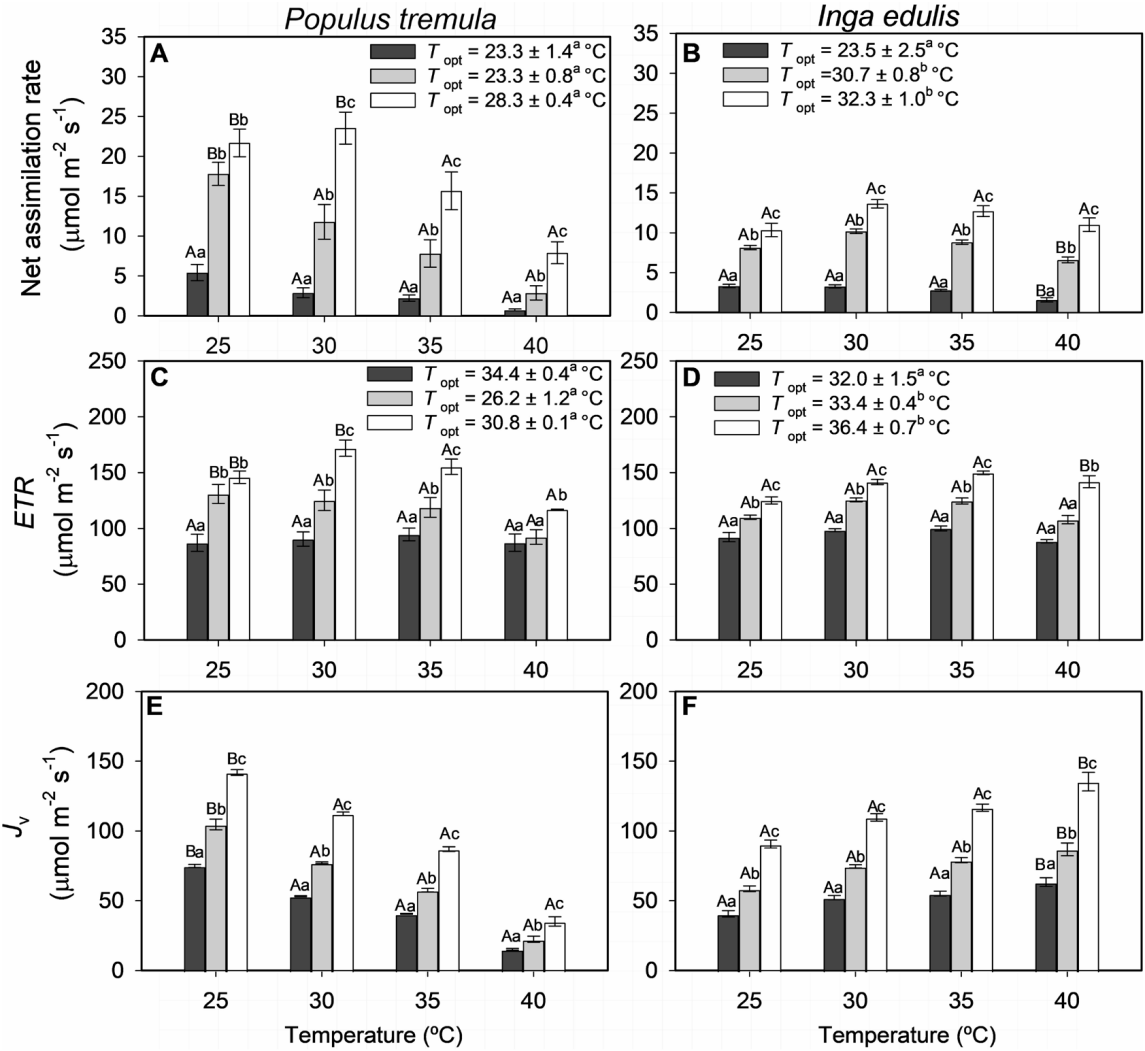
Temperature response of net assimilation rate (A, B), electron transport rate (*ETR*) (C, D), and electron flux used for the carboxylation (*J*_v_) (E, F) in *Populus tremula* (aspen) and *Inga edulis* (ice‐cream‐bean, ingá‐cipó) under three CO_2_ concentrations: 150 μmol mol^−1^ (black), 400 μmol mol^−1^ (gray), and 1000 μmol mol^−1^ (white). Panels on the left (A, C, E) correspond to *Populus tremula*, and those on the right (B, D, F) to *Inga edulis* . Data were fitted using an Arrhenius‐type model (Equation 2), and the values of the estimated optimum temperature (*T*_opt_, Equation 3) are indicated (means ± SE) for each CO_2_ concentration. *T*_opt_ values were compared between species, and different letters indicate significant differences at *p* < 0.05. Different lowercase letters indicate significant differences between CO_2_ treatments at the same temperature (*p* < 0.05), and uppercase letters compare differences between species at the same temperature and CO_2_ concentration. Means ± SE are based on 3–9 replicate leaves per treatment.

For both *Populus tremula* and *Inga edulis* , increasing CO_2_ levels had a significantly positive effect on net assimilation (*A*). However, temperature influenced the species differently (Figure 1A,B). In *P. tremula* , temperature had a strong negative impact on *A*, with a significant negative interaction with CO_2_ (slope ± SE of −0.0007 ± 0.0003 μmol m^−2^ s^−1^ per μmol mol^−1^ per °C, Table 1). This effect intensified as temperatures rose, especially noticeable at 40°C, though the decline was moderated at higher CO_2_ concentrations (1000 μmol mol^−1^) (Figure 1A). Conversely, *I. edulis* exhibited stable *A* levels across temperature variations, particularly under high CO_2_, indicating a lack of significant temperature sensitivity (Figure 1B, Table 1).

**TABLE 1:** Regression coefficients (intercept and slope) and their corresponding standard errors for the effects of CO_2_, temperature, and their interaction (CO_2_ × temperature) on various physiological variables of *Populus tremula* and *Inga edulis* .

Species	Dependent variable	Intercept	Std. Error	Independent variable						
				CO_2_		Temperature		Interaction CO_2_ × temperature		*r* ^2^
				Slope	Std. Error	Slope	Std. Error	Slope	Std. Error	
*Populus tremula*	*A* (μmol m^−2^ s^−1^)	26.2***	3.4	0.016***	0.002	−0.75***	0.10	−0.0007*	0.0003	0.84
	*ETR* (μmol m^−2^ s^−1^)	143.9***	14.3	0.066***	0.007	−1.83***	0.42	−0.0033**	0.0012	0.80
	*J*_v_ (μmol m^−2^ s^−1^)	214.3***	18.7	0.055***	0.009	−5.36***	0.55	−0.0033*	0.0015	0.81
	*I* (nmol m^−2^ s^−1^)	−95.7***	6.4	0.002^ns^	0.003	4.37***	0.19	0.0012*	0.0005	0.94
	DMADP (nmol m^−2^)	−1975**	689	0.196 ^ns^	0.318	114.2***	20.3	0.086^ns^	0.057	0.52
	*ETR*‐*J*_v_	−70.3***	16.4	0.011 ^ns^	0.008	3.52***	0.48	0.00009 ^ns^	0.00136	0.63
*Inga edulis*	*A* (μmol m^−2^ s^−1^)	0.42**	1.57	0.0101***	0.0007	−0.052^ns^	0.046	0.0002^ns^	0.0001	0.78
	*ETR* (μmol m^−2^ s^−1^)	82.3***	8.2	0.050***	0.004	0.28^ns^	0.24	0.0017*	0.0007	0.77
	*J*_v_ (μmol m^−2^ s^−1^)	−21.1**	6.2	0.070***	0.003	2.01***	0.18	0.0017**	0.0005	0.93
	*I* (nmol m^−2^ s^−1^)	−56.8***	3.5	−0.017***	0.104	3.04***	0.10	−0.0013***	0.0003	0.95
	DMADP (nmol m^−2^)	−1743***	350	−0.58***	0.16	120.4***	10.3	0.042 ^ns^	0.029	0.73
	*ETR*‐*J*_v_	103.4***	7.9	−0.0197***	0.0036	−1.73***	0.23	0.00004^ns^	0.00065	0.60

*Note:* The dependent variables include net assimilation rate (*A*), electron transport rate (*ETR*), electron transport rate supporting carbon assimilation (*J*_v_), isoprene emission rate (*I*), dimethylallyl diphosphate pool size (DMADP), and the electron flux not used for the carboxylation, calculated as the difference between *ETR* and *J*_v_ (*ETR*‐*J*_v_). Asterisks indicate the level of statistical significance (*p < 0.05; **p < 0.01; ***p < 0.001), while “ns” denotes non‐significant results. The *r* ^2^ values represent the proportion of variance explained by the regression models.

This disparity in temperature response led to differences in optimal temperature (*T*_opt_, Equation 3) for photosynthesis between species. Under high CO_2_, *P. tremula* exhibited a lower *T*_opt_ (28.3°C ± 0.4°C) compared to *I. edulis* (32.3°C ± 1.0°C). Across the 25°C–40°C range, the average activation energy (Δ*H*_a_, Equation 2) and associated *Q*_10_ values (Equation 4) showed distinct species‐specific patterns: in *P. tremula* , both Δ*H*_a_ and *Q*_10_ decreased with increasing CO_2_, whereas in *I. edulis* , both parameters rose. However, the overall Δ*H*_a_ and *Q*_10_ for *A* did not significantly differ between species (Table 2).

**TABLE 2:** Activation energy, Δ*H*_a_ (Equation 2) and *Q*_10_ (Equation 4) values for net assimilation rate (*A*), electron transport rate (*ETR*), isoprene emission rate (*I*), dimethylallyl diphosphate pool size (DMADP), and isoprene synthase (IspS) activity in *Populus tremula* and *Inga edulis* at different CO_2_ concentrations (150, 400, and 1000 μmol mol^−1^) over a measurement temperature range of 25°C–40°C.

Species	CO_2_ concentration (μmol mol^−1^)	*A* (μmol m^−2^ s^−1^)	*ETR* (μmol m^−2^ s^−1^)	*I* (nmol m^−2^ s^−1^)	DMADP (nmol m^−2^)	IspS activity (s^−1^)
Δ*H*_a_ (KJ mol^−1^)						
*Populus tremula*	150	75.0 ± 17.8^a^	19.1 ± 6.8^a^	96.3 ± 3.8^a^	134.0 ± 8.2^a^	60.2 ± 1.8^a^
	400	78.4 ± 2.8^a^	16.6 ± 1.5^a^	111.3 ± 4.6^b^	116.8 ± 11.9^a^	62.6 ± 1.9^a^
	1000	47.3 ± 7.7^a^	37.2 ± 2.5^b^	136.5 ± 6.3^b^	132.9 ± 3.1^a^	58.8 ± 0.7^b^
*Inga edulis*	150	48.7 ± 20.4^a^	23.4 ± 6.8^a^	90.6 ± 1.8^a^	78.6 ± 15.2^a^	73.2 ± 12.6^a^
	400	55.6 ± 10.0^a^	30.1 ± 7.6^a^	84.5 ± 1.9^a^	93.8 ± 16.4^a^	54.1 ± 12.3^a^
	1000	73.4 ± 15.4^a^	22.3 ± 3.2^a^	99.5 ± 6.8^a^	132.3 ± 15.0^a^	29.3 ± 4.0^a^
*Q*_10_						
*Populus tremula*	150	2.73 ± 0.40^a^	1.28 ± 0.08^a^	3.34 ± 0.14^a^	5.41 ± 0.43^b^	2.12 ± 0.05^a^
	400	2.67 ± 0.08^a^	1.24 ± 0.02^a^	4.03 ± 0.20^b^	4.45 ± 0.55^a^	2.19 ± 0.05^a^
	1000	1.83 ± 0.13^a^	1.61 ± 0.04^b^	5.55 ± 0.39^b^	5.27 ± 0.22^a^	2.08 ± 0.03^b^
*Inga edulis*	150	2.22 ± 0.70^a^	1.37 ± 0.12^a^	3.10 ± 0.09^a^	2.90 ± 0.50^a^	2.66 ± 0.44^a^
	400	2.08 ± 0.27^a^	1.49 ± 0.14^a^	2.88 ± 0.09^a^	3.53 ± 0.59^a^	2.09 ± 0.33^a^
	1000	2.76 ± 0.59^a^	1.33 ± 0.05^a^	3.53 ± 0.35^a^	5.74 ± 1.11^a^	1.45 ± 0.07^a^

*Note:* Data are presented as means ± SE of three to five independent samples (trees). Means were compared using ANOVA, and different letters indicate statistically significant differences between the species at each CO_2_ concentration (*p* < 0.05).

The temperature sensitivity difference between *P. tremula* and *I. edulis* was even more pronounced in the electron transport rate (*ETR*) (Figure 1C,D). In *P. tremula* , *ETR* exhibited a marked decline with increasing temperature, as indicated by a significant slope of −1.83 ± 0.42 μmol m^−2^ s^−1^ per °C. Additionally, *ETR* showed a negative interaction with CO_2_, with a slope of −0.0033 ± 0.0012 μmol m^−2^ s^−1^ per μmol mol^−1^ (Table 1), indicating that elevated CO_2_ does not mitigate the temperature‐induced inhibition of *ETR*. This decrease in *ETR* was also associated with a pronounced reduction in electron flux for carboxylation (*J*_v_) at higher temperatures, reflecting a similar negative interaction with CO_2_ (Figure 1E, Table 1). This sensitivity aligns with the higher Δ*H*_a_ and *Q*_10_ values observed for *P. tremula* under elevated CO_2_ (Table 2), indicating a steeper temperature dependence for its electron transport.

At 25°C and 30°C, *P. tremula* displayed significantly higher *ETR* values under elevated CO_2_ compared to *I. edulis* . For instance, at 30°C and 1000 μmol mol^−1^ CO_2_, the mean *ETR* for *P. tremula* was 171.5 ± 7.3 μmol m^−2^ s^−1^ and for *I. edulis* was 141.6 ± 2.3 μmol m^−2^ s^−1^ (Figure 1C,D). Similarly, at 25°C, the *J*_v_ values for *P. tremula* were significantly higher, with an overall mean of 107.2 μmol m^−2^ s^−1^ compared to 63.3 μmol m^−2^ s^−1^ in *I. edulis* (Figure 1E,F).

As temperature increased to 40°C, *I. edulis* exhibited significantly higher *ETR* and *J*_v_ values across all CO_2_ concentrations. For *ETR*, *I. edulis* reached a mean of 141.8 ± 5.3 μmol m^−2^ s^−1^ under high CO_2_, while *P. tremula* declined sharply to 117.0 ± 0.4 μmol m^−2^ s^−1^. Similarly, *J*_v_ in *I. edulis* reached 135.3 ± 6.6 μmol m^−2^ s^−1^ at 1000 μmol mol^−1^, while in *P. tremula* it was 35.1 μmol m^−2^ s^−1^. The largest relative increase in *J*_v_ occurred at the lowest CO_2_ concentration, where *J*_v_ in *I. edulis* was 4.2 times greater than in *P. tremula* .

In *I. edulis* , temperature alone did not significantly impact *ETR* (*p* > 0.05). However, the interaction with elevated CO_2_ produced a mild but positive effect on *ETR* (slope = 0.0017 ± 0.0007 μmol m^−2^ s^−1^ per μmol mol^−1^ per °C, Table 1), with a steeper increase in *ETR* under high CO_2_ and temperature (Figure 1D). This positive interaction effect was even more pronounced in *J*_v_ (Figure 1F), where the combination of increased temperature and CO_2_ significantly enhanced flux rates (Figure 1F, Table 1; see Figure S1 for species comparisons). The lower Δ*H*_a_ and *Q*_10_ values indicate a more temperature‐stable *ETR* response in *I. edulis* under elevated CO_2_ (Table 2). As a result, *I. edulis* exhibited a higher *T*_opt_ for *ETR* under elevated CO_2_ (36.4°C ± 0.7°C), compared to *P. tremula* (30.8°C ± 0.1°C) under the same conditions (Figure 1C,D).

### 3.2 Species‐Specific Dependencies of Isoprene Emission on Temperature and [CO_2_ ] Interactions in *Populus tremula* and *Inga edulis*

**FIGURE 2:**
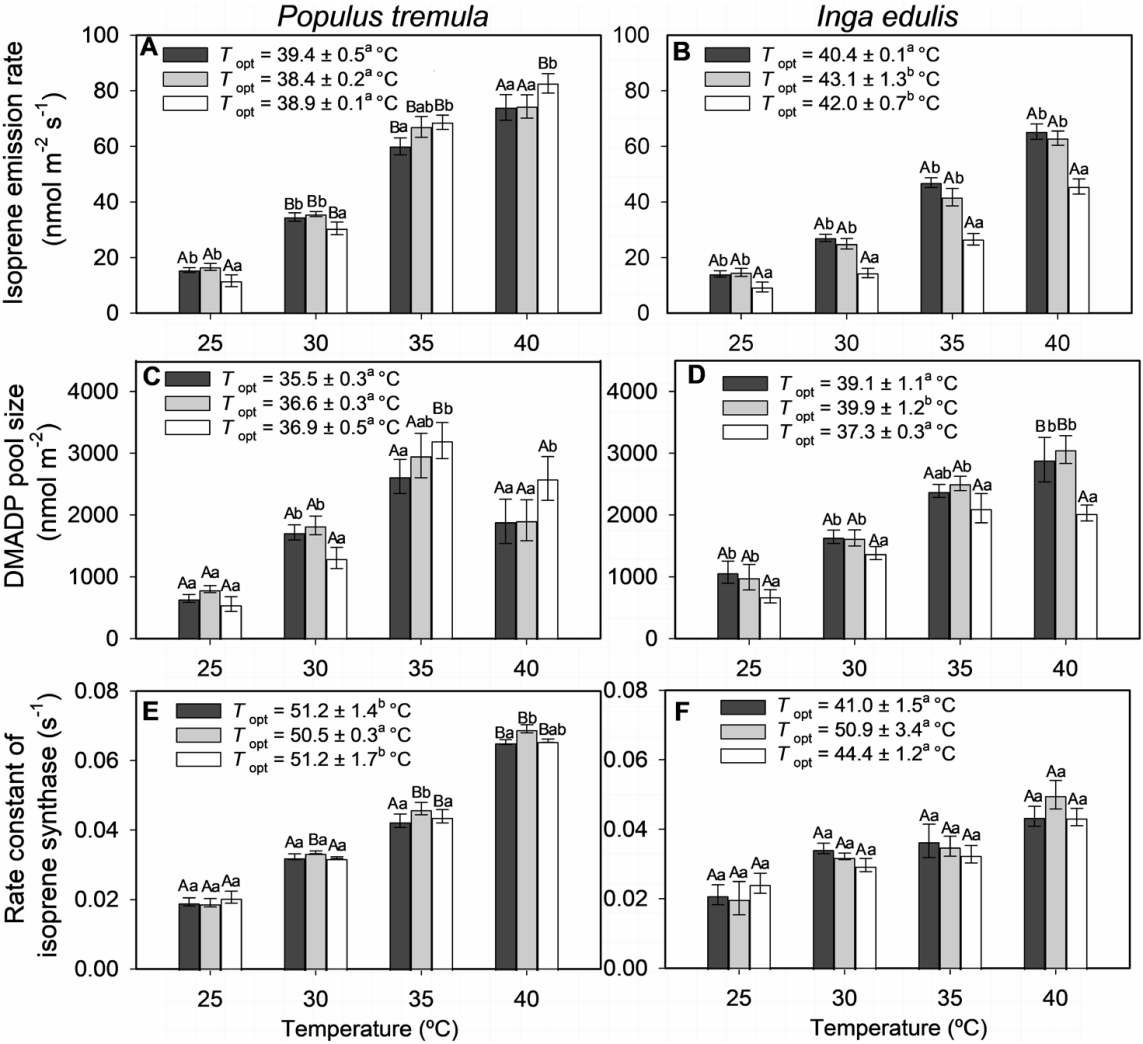
Temperature response of isoprene emissions rate (A, B), DMADP pool size (C, D), and isoprene synthase activity (E, F) in *Populus tremula* and *Inga edulis* under three CO_2_ concentrations: 150 μmol mol^−1^ (black), 400 μmol mol^−1^ (gray), and 1000 μmol mol^−1^ (white). Data presentation, statistical significance, and replicates are described in Figure 1.

In *P. tremula* , suppression of isoprene emissions by elevated CO_2_ was observed only at lower temperatures (25°C and 30°C). The lowest emission rate (11.7 ± 2.1 nmol m^−2^ s^−1^) was recorded at 25°C and 1000 μmol mol^−1^ CO_2_ (Figure 2A). As temperature increased to 35°C, the inhibitory effect of CO_2_ dissipated, and emissions rose, peaking at 82.7 ± 3.5 nmol m^−2^ s^−1^ at 40°C under elevated CO_2_. The calculated *T*_opt_ for isoprene emission was slightly lower, around 38°C, indicating that maximal efficiency occurred just below the observed peak. Although the isolated effect of CO_2_ on isoprene emissions was not statistically significant, the positive interaction between temperature and CO_2_ was (slope = 0.0012 ± 0.0005 nmol m^−2^ s^−1^ per μmol mol^−1^ per °C, Table 1).

In contrast, *I. edulis* consistently showed suppression of isoprene emissions under high CO_2_ across all temperatures (Figure 2B). The maximum emission rate (65.3 ± 2.7 nmol m^−2^ s^−1^) occurred at 40°C under sub‐ambient CO_2_, while the minimum rate (9.4 ± 1.8 nmol m^−2^ s^−1^) occurred at 25°C under elevated CO_2_. The negative interaction effect of temperature and CO_2_ concentration was observed (slope = −0.0013 ± 0.0003 nmol m^−2^ s^−1^ per μmol mol^−1^ per °C). Isoprene emissions increased with temperature under all CO_2_ levels; however, the magnitude of this increase was lower at 1000 μmol mol^−1^ compared to 150 or 400 μmol mol^−1^, particularly at higher temperatures. Figure S2B further confirms this trend, showing reduced temperature sensitivity under high CO_2_, with lower slopes and *r* ^2^ values.

The temperature sensitivity of isoprene emissions also differed significantly between the species. In *P. tremula* , Δ*H*_a_ for isoprene emissions increased with rising CO_2_, from 96.3 ± 3.8 kJ mol^−1^ at 150 μmol mol^−1^ CO_2_ to 136.5 ± 6.3 kJ mol^−1^ at 1000 μmol mol^−1^ CO_2_. This increase was reflected in higher *Q*_10_ values, indicating greater thermal sensitivity under elevated CO_2_ (Table 2). Similarly, in *I. edulis* , Δ*H*_a_ increased from 90.6 ± 1.8 kJ mol^−1^ to 99.5 ± 6.8 kJ mol^−1^, aligning with its stable isoprene emissions rates across a broader temperature range. Despite the smaller magnitude of increase in Δ*H*_a_ for *I. edulis* , the higher Δ*H*_a_ and *Q*_10_ values observed in *P. tremula* suggest a steeper temperature dependence for isoprene emission in this species, particularly under elevated CO_2_. Despite the lower Δ*H*_a_, *I. edulis* maintained a higher *T*_opt_ for isoprene emission, estimated at approximately 42°C–43°C under both 400 and 1000 μmol mol^−1^ CO_2_, compared to 38°C in *P. tremula* at similar CO_2_ levels (Figure 2A,B). This analysis indicates that while the highest emissions were observed experimentally at 40°C, *I. edulis* achieves maximal efficiency at slightly higher temperatures.

In both species, the *T*_opt_ for isoprene emission exceeded that for *A* (Figure 1A,B) and *ETR* (Figure 1C,D), indicating that isoprene synthesis operates optimally at higher temperatures than photosynthetic processes.

### 3.3 Characterization of Isoprene Emission Responses to Temperature and CO_2_ Concentrations

**FIGURE 3:**
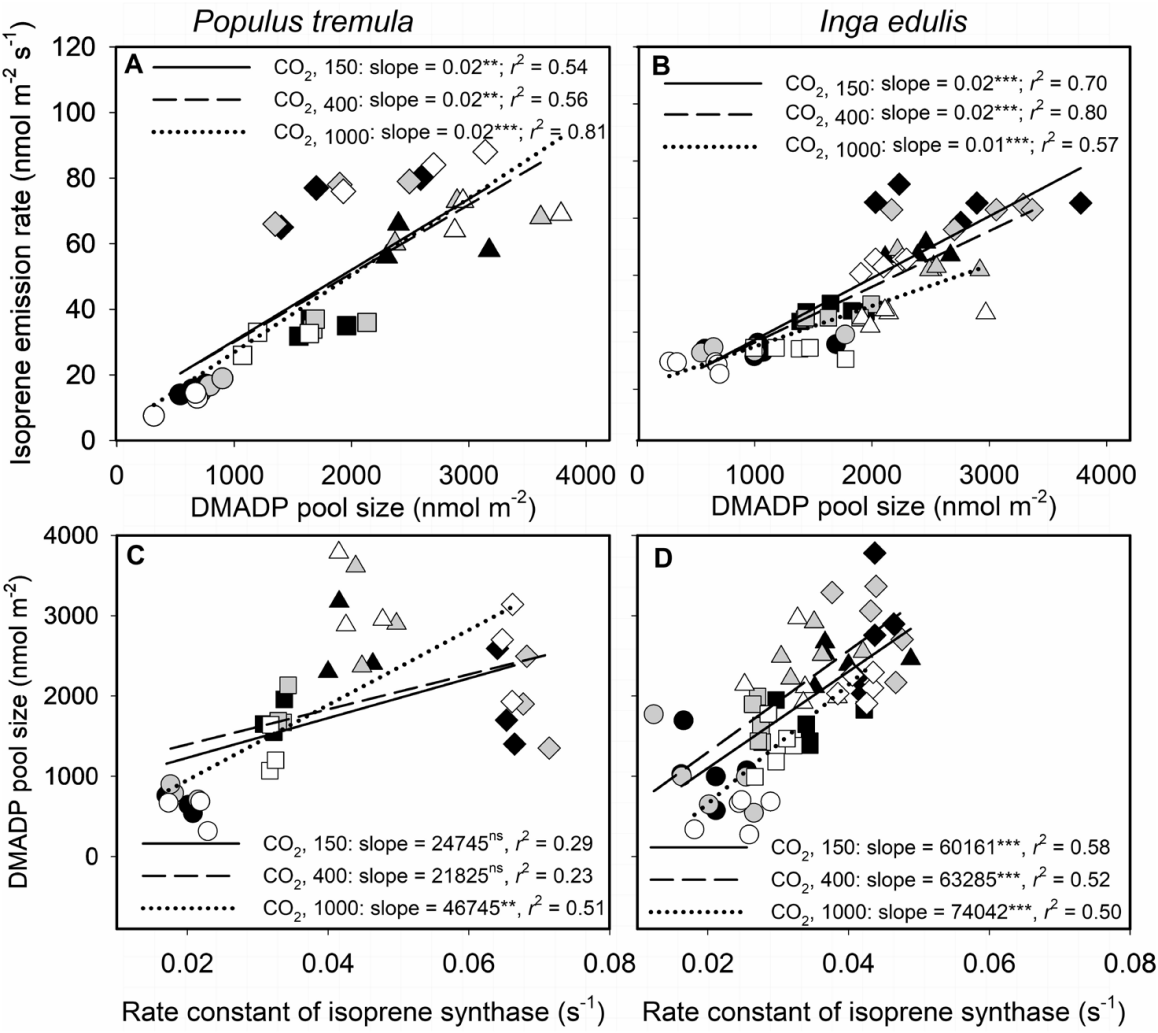
Relationships between isoprene emission rate and DMADP pool size (A, B), and DMADP pool size and rate constant of isoprene synthase (C, D) across temperatures at different CO_2_ concentrations. Panels on the left (A and C) correspond to *Populus tremula*, and those on the right (B and D) to *Inga edulis* . The data were fitted by linear regression for each individual CO_2_ concentration across temperatures. Black, gray, and white symbols represent CO_2_ concentrations of 150, 400, and 1000 μmol mol^−1^, respectively. Different shapes correspond to the four temperatures: Circles for 25°C, squares for 30°C, triangles for 35°C, and diamonds for 40°C. Solid, dashed, and dotted regression lines correspond to 150, 400, and 1000 μmol mol^−1^ CO_2_, respectively. Coefficients of determination (*r* ^2^) and the significance of slopes are provided. Asterisks indicate levels of statistical significance (*p < 0.05; **p < 0.01; ***p < 0.001), and “ns” denotes non‐significant results.

In *P. tremula* , high CO_2_ reduced the DMADP pool size at lower temperatures but lost this effect as temperature increased from 30°C to 35°C. DMADP level peaked at 3205 ± 291 nmol m^−2^ under 1000 μmol mol^−1^ CO_2_, with *T*_opt_ of 36.9°C ± 0.5°C (Figure 2C). Beyond this temperature, DMADP levels declined, though the reduction was less pronounced under 1000 μmol mol^−1^ compared to 150 and 400 μmol mol^−1^. The decline in DMADP above *T*_opt_ was less steep under elevated CO_2_, aligning with the pattern of higher isoprene emission at 1000 μmol mol^−1^ CO_2_ (Figure 3A, see also Figure S2C).

In contrast, *I. edulis* exhibited consistent suppression of DMADP pool size by elevated CO_2_ across all temperature ranges (Figure 2D). Both CO_2_ and temperature significantly affected DMADP levels, with a strong negative effect (slope = −0.58 ± 0.16 nmol m^−2^ per μmol mol^−1^, *p* < 0.001) and a positive temperature effect (slope = 120.4 ± 10.3 nmol m^−2^ per °C, *p* < 0.001; Table 1). As temperatures rose from 25°C to 40°C, both isoprene emission and DMADP pool size increased. However, suppression by elevated CO_2_ persisted, maintaining the DMADP‐isoprene relationship (Figure 3B). Under ambient CO_2_, *I. edulis* exhibited a higher *T*_opt_ for DMADP than *P. tremula* (Figure 2C,D).

For DMADP dynamics, the average Δ*H*_a_ and associated *Q*_10_ values exhibited species‐specific trends across CO_2_ levels. In *P. tremula* , Δ*H*_a_ increased modestly from 116.8 ± 11.9 kJ mol^−1^ under ambient CO_2_ to 132.9 ± 3.1 kJ mol^−1^ under elevated CO_2_ (Table 2), corresponding to *Q*_10_ values of 4.45 ± 0.55 kJ mol^−1^ and 5.27 ± 0.22 kJ mol^−1^, respectively. Conversely, in *I. edulis* , Δ*H*_a_ increased more substantially, from 93.8 ± 16.4 kJ mol^−1^ under ambient CO_2_ to 132.3 ± 15.0 kJ mol^−1^ at elevated CO_2_. This pattern resulted in similar Δ*H*_a_ values between species at elevated CO_2_, despite the lower baseline observed in *I. edulis* . Despite these trends, statistical comparisons showed no significant species‐level differences in Δ*H*_a_ and *Q*_10_ for DMADP (*p* > 0.05).

Isoprene synthase (IspS) activity increased steadily with temperature in both species, showing no significant effect from CO_2_ nor its interaction with temperature (Figure 2E,F). Temperature effects on IspS activity were more pronounced in *P. tremula* (see Figure S2E,F), with a *T*_opt_ exceeding 50°C—a significant difference under both 150 and 1000 μmol mol^−1^ CO_2_ (Figure 2E,F). At 40°C, maximum IspS activity reached 0.069 ± 0.001 s^−1^ in *P. tremula* and 0.050 ± 0.004 s^−1^ in *I. edulis* .

In *P. tremula* , no correlation was observed between IspS activity and DMADP under 150 and 400 μmol mol^−1^ CO_2_ (Figure 3C). However, a correlation emerged under 1000 μmol mol^−1^ CO_2_, reflecting the strengthened coupling between isoprene emission and DMADP at high CO_2_ (Figure 3A). Conversely, in *I. edulis* , a consistent correlation between IspS activity and DMADP pool size was observed across all CO_2_ concentrations and temperatures (Figure 3D). Notably, this correlation was weaker than that between isoprene emission and DMADP, indicating distinct dynamics of IspS activity and its role in isoprene synthesis in the two species.

### 3.4 Relationship Between Electron Flux Partitioning, Photosynthesis, and Isoprene Emission in *Populus tremula* and *Inga edulis*

**FIGURE 4:**
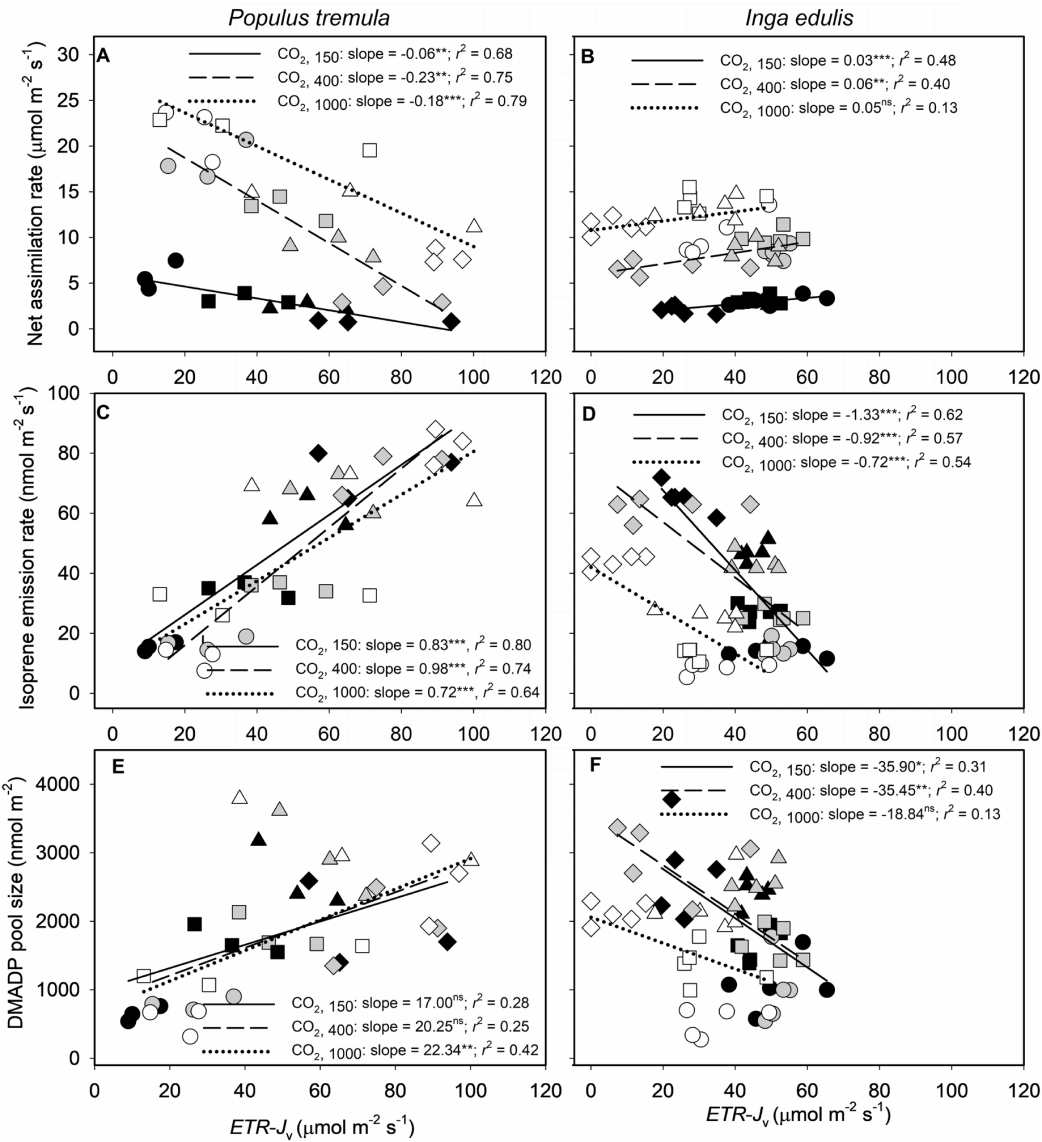
Relationship between net assimilation rate (A, B), isoprene emission rate (C, D), and DMADP pool size (E, F) with electron flux not used for carboxylation in *P. tremula* and *I. edulis* . The electron flux not used for carboxylation is calculated as the difference between the rate of electron transport (*ETR*) and the electron flux required for carboxylation (*J*_v_, Equation 1). Data presentation and statistical analysis are described in Figure 3.

In *P. tremula* , *A* was negatively correlated with the electron transport rate directed toward carboxylation (*ETR*‐*J*_v_) as temperature increased across all CO_2_ levels. This decline was more pronounced under ambient and high CO_2_ conditions (Figure 4A). In contrast, *I. edulis* displayed minimal sensitivity to *ETR*‐*J*_v_ changes, with a slight positive correlation between *A* and *ETR*‐*J*_v_ (Figure 4B). Notably, at lower CO_2_ levels, *I. edulis* showed increased *A* as temperature decreased, aligning with a relative rise in *ETR*‐*J*_v_. Under high CO_2_, the temperature had no significant impact on *A*, except for a slight decrease at 25°C, where *ETR*‐*J*_v_ reached a moderate value, indicating a stable assimilation pattern across varying *ETR*‐*J*_v_ levels.

Isoprene emission rates further highlighted distinctions between *P. tremula* and *I. edulis* . In *P. tremula* , emissions increased significantly with *ETR*‐*J*_v_, particularly as temperature and CO_2_ levels rose (Figure 4C). This trend suggests that the diminished CO_2_ sensitivity at higher temperatures was associated with elevated energetic demands from electron transport. Additionally, DMADP pool size in *P. tremula* was positively correlated with *ETR*‐*J*_v_ at elevated CO_2_ concentration and temperature (Figure 4E). However, peak DMADP levels at 35°C did not coincide with peak electron flux toward non‐carboxylative pathways (Figure 2E), suggesting a decoupling between precursor availability and flux direction under these conditions. At 40°C, DMADP levels in *P. tremula* declined as electron flux shifted toward alternative energy sinks rather than CO_2_ assimilation. Despite this reduction in DMADP, the isoprene emission rate continued to rise due to the direct temperature‐driven increases in IspS activity (Figure 3A).

In *Inga edulis* , an inverse relationship between isoprene emission and *J*_v_ was observed, particularly under high CO_2_ and low temperatures (Figure 4D). At lower temperatures, electron flux shifted to non‐carboxylative sinks without a corresponding increase in isoprene emissions (Figure 4D). Under elevated CO_2_, *I. edulis* exhibited a tendency for electron allocation toward carboxylation, which was associated with consistently lower DMADP levels across all temperatures (Figure 4D,F). In contrast, *P. tremula* demonstrated greater redistribution of electron flux under elevated CO_2_ and temperature stress, maintaining isoprene synthesis even as DMADP levels declined (Figure 4E).

## 4 Discussion

### 4.1 Thermotolerance Threshold Dictates the CO_2_ Suppression on Isoprene Biosynthesis via Substrate Limitations

This study highlights distinct thermotolerance thresholds and CO_2_ suppression responses in *I. edulis* and *P. tremula* , with each species exhibiting unique adaptations in photosynthetic efficiency, electron flux partitioning, and isoprene emission. *I. edulis* demonstrated higher *T*_opt_ for both *A* (32.3°C ± 1.0°C) and *ETR* (36.4°C ± 0.7°C) and a broader tolerance to CO_2_ suppression across temperature ranges, aligning with its adaptation to high light and nutrient‐limited tropical environments (Cunningham and Read 2002; Manu et al. 2024). In contrast, *P. tremula* exhibited lower *T*_opt_ values for *A* (28.3°C ± 0.4°C) and *ETR* (30.8°C ± 0.1°C) and a more restricted range of CO_2_ suppression on isoprene emission (see Table 1). This may result from the decreased stability of photosynthetic membranes and a reduction in photosynthetic efficiency under heat stress (Arab et al. 2016; Niinemets 2018; Zhu et al. 2018).

Although these species were not grown under identical environmental conditions, their contrasting responses align with thermal strategies shaped by their native biomes. However, whether these differences arise from inherent traits or reflect plastic responses to growth conditions remains uncertain. Studies combining field and controlled environments suggest that both acclimation and inherent differences influence photosynthetic heat tolerance. For example, Zhu et al. (2018) showed that species from warmer biomes exhibit higher photosynthetic temperature optima than temperate species, even under identical conditions. More broadly, temperature sensitivity in photosynthesis tends to follow biogeographic patterns, increasing linearly by approximately 8°C from polar to equatorial regions (O'sullivan et al. 2017). Similarly, Crous et al. (2022) showed that *T*_opt_ for *A* increases predictably with warming across evergreen species from boreal to tropical biomes, reinforcing the role of both acclimation and adaptation. While our study includes only two species, they were deliberately selected to represent tropical and temperate extremes, offering mechanistic insights into how native thermal environments may shape energy allocation and isoprene metabolism. These findings support the view that some of the observed variation reflects physiological specialization, though standardized experiments are needed to disentangle the roles of plasticity and inherent species‐level differences.

CO_2_‐induced suppression of isoprene emission rates was most pronounced near each species' *T*_opt_ for *A* and *ETR*, closely associated with a reduction in DMADP pool size. This suppression likely arises from energy limitations within the chloroplast under high CO_2_, where increased ATP and NADPH demands for carbon assimilation constrain resources available for isoprene biosynthesis (Niinemets et al. 1999b; Morfopoulos et al. 2014; Dani et al. 2014; Rasulov et al. 2016). Given *I. edulis* 's higher *T*_opt_, elevated temperatures under high CO_2_ conditions gradually enhanced *ETR*, facilitating continuous electron flux toward carbon assimilation at the expense of other energy sinks, including isoprene emission. As a result, *I. edulis* exhibited a broader range of CO_2_ suppression on isoprene emission, extending up to 40°C. This suppression was closely tied to reductions in DMADP pool size and sustained electron flux toward carboxylation, especially under elevated CO_2_ conditions (Figure 4C,D). This trend, along with a temperature‐driven increase in IspS activity (Figure 1F), underscores the interplay between CO_2_ inhibition and electron flux partitioning to carboxylation pathways.

Interestingly, at lower temperatures, *I. edulis* displayed increased sensitivity in energy fluxes, with greater shifts toward non‐carboxylative pathways and concurrent reductions in isoprene emissions and DMADP pool size. These observations align with studies suggesting that, in warm‐climate species, low temperatures disproportionately affect biochemical processes related to photosynthesis compared to light harvesting and energy transfer (Allen and Ort 2001; Lambers et al. 2008). This differential sensitivity likely drives increased electron flux toward non‐photosynthetic carbon sinks, accompanied by reduced MEP pathway activity at low temperatures.

In contrast, *P. tremula* exhibited greater sensitivity to elevated temperatures, with significant energetic imbalances as temperature increased. These imbalances diminished CO_2_ suppression effects on isoprene emission and DMADP pool size, driven by enhanced electron transport rates toward non‐photosynthetic carbon reduction sinks (*ETR*‐*J*_v_, Figure 4A) and a likely increase in the availability of reducing power for the MEP pathway (Niinemets et al. 1999b; Morfopoulos et al. 2014; Dani et al. 2014; Rasulov et al. 2016). Given that isoprene synthesis requires relatively little ATP and NADPH compared to carbon assimilation, it often relies on excess reducing power (Jardine et al. 2014; Dani et al. 2015; Rasulov et al. 2016). This excess reducing power becomes more available when carbon reduction capacity decreases, resulting in higher DMADP levels and increased isoprene emission rates (Rasulov et al. 2009). These dynamics explain why CO_2_ suppression on isoprene emission intensifies within each species' *T*_opt_ range for photosynthesis, where demand by ATP and NADPH for carbon assimilation increases, limiting resources for other energy sinks, including isoprene biosynthesis (Niinemets et al. 1999b; Selmar and Kleinwächter 2013; Dani et al. 2015). Despite this, *P. tremula* exhibited peak isoprene emissions at 40°C under 1000 μmol mol^−1^ CO_2_, even as DMADP pool decreased—a response likely driven by enhanced IspS activity (Figure 3A,C). While heat stress disrupts key enzymes within the MEP pathway, leading to reduced DMADP production at temperatures above 35°C (Rasulov et al. 2010; Li et al. 2011), the IspS in *P. tremula* has a higher *T*_opt_ than photosynthesis processes and MEP enzymes (Li et al. 2011; Ghirardo et al. 2014), enabling partial compensation for substrate limitations under thermal stress. This interaction between substrate availability and enzyme kinetics suggests that isoprene emissions at high temperatures are regulated by the thermal sensitivity of IspS and bottlenecks within the MEP pathway (Rivasseau et al. 2009; Rasulov et al. 2010; Ghirardo et al. 2014; Sahu et al. 2023).

### 4.2 Comparative *Q*_10_ and Δ*H*_a_ for *ETR* and Isoprene Emission

Although both species displayed similar Δ*H*_a_ and *Q*_10_ values for *A* (Table 2), *P. tremula* exhibited higher Δ*H*_a_ and *Q*_10_ values for *ETR* under high CO_2_. This indicates stronger temperature dependence for electron transport, potentially contributing to rapid increases in photosynthesis within its optimal range but also increased vulnerability to heat stress as temperatures exceed *T*_opt_. Additionally, the *Q*_10_ for isoprene emission in *P. tremula* was notably higher than for CO_2_ assimilation, reflecting greater temperature sensitivity for isoprene synthesis. This pattern aligns with findings by Sahu et al. (2023), which demonstrated significantly higher *Q*_10_ values for isoprene emission (*Q*_10_ = 10.3 at 78 Pa CO_2_ vs. *Q*_10_ = 4.6 at 41 Pa CO_2_) compared to photosynthesis (*Q*_10_ = 1.2) under elevated CO_2_ conditions. Although *I. edulis* also displayed a higher *Q*_10_ for isoprene emission than for *A*, the difference was smaller than that observed in *P. tremula* , especially under high CO_2_ (see Table 2). This pattern suggests that isoprene emissions in *P. tremula* are more temperature‐sensitive than photosynthesis, particularly under high CO_2_, likely due to enhanced IspS activity acting as a mechanism to dissipate excess reducing power under heat stress. In contrast, *I. edulis* maintains a relatively stable balance between isoprene emission and CO_2_ assimilation, which may contribute to its greater thermal tolerance and stability across a range of CO_2_ levels.

### 4.3 Electron Flux Allocation and Its Role in Temperature‐Driven Isoprene Emission Responses

**FIGURE 5:**
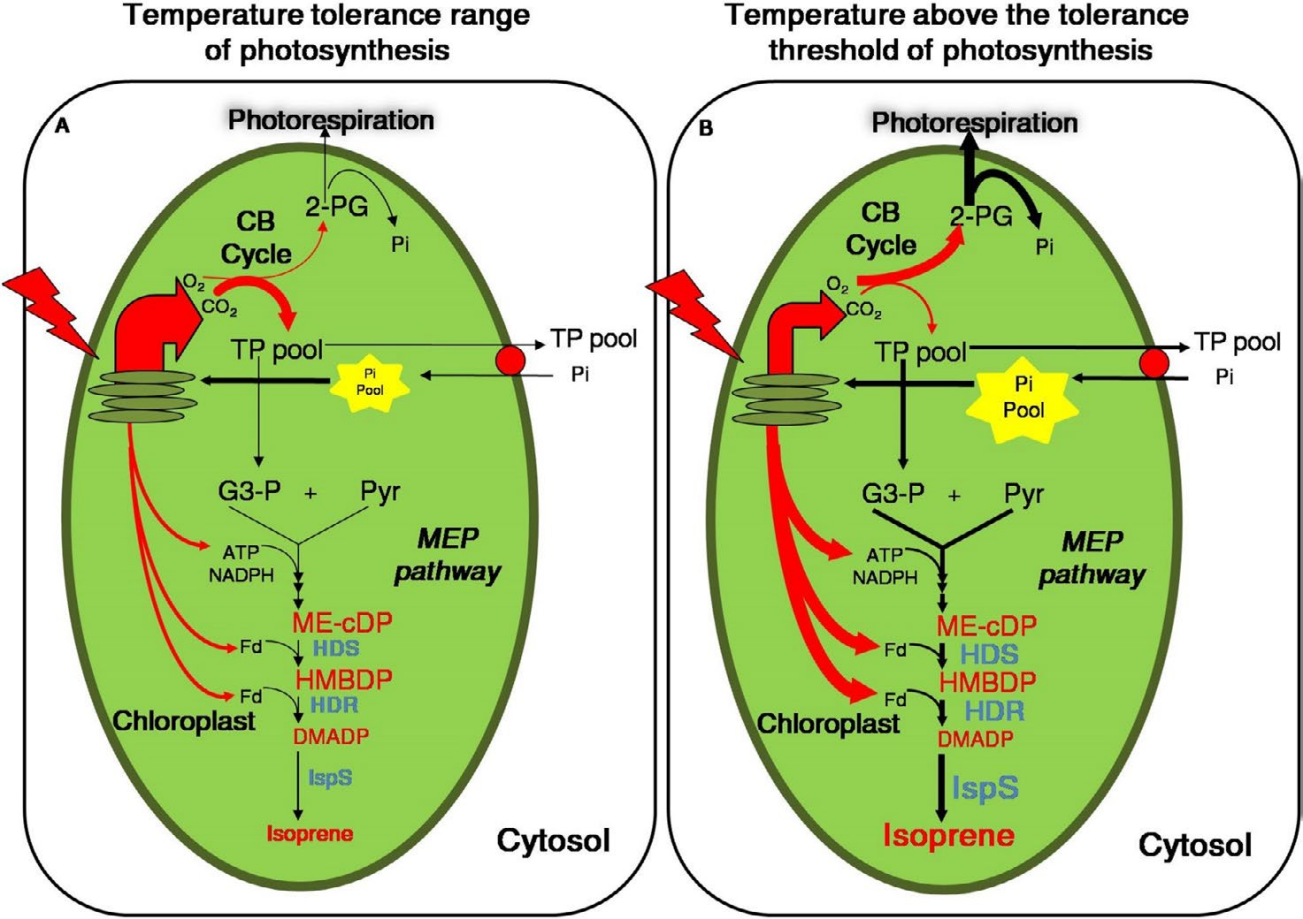
Schematic representation of electron partitioning between net CO_2_ assimilation and isoprene synthesis under elevated CO_2_ concentrations ([CO_2_]), considering species‐specific temperature tolerance thresholds. (A) Within the temperature tolerance range for photosynthesis, species adapted to high physiological temperatures increase the demand for reducing power and ATP through the Benson‐Calvin (CB) cycle when exposed to elevated [CO_2_]. This results in a reduced allocation of electrons to isoprene synthesis. (B) When the temperature exceeds the photosynthetic tolerance threshold, excess energy accumulates in the light reaction components, increasing the supply of reducing power for DMADP production, which subsequently supports isoprene synthesis. The enzymes HDS (HMBDP synthase) and HDR (HMBDP reductase) accept electrons directly from reduced ferredoxin (*F*_d_), thereby facilitating the production of DMADP and isoprene. During short high‐temperature events, maintaining the transthylakoid potential and reducing carboxylation rates helps sustain additional reducing power for MEP pathway enzyme activity. Under heat stress, however, the electron transport chain may be impaired, reducing the availability of chloroplastic reductants and potentially limiting the production of MEP intermediates for isoprene biosynthesis. At lower temperatures, inorganic phosphate (P_i_) content is reduced, which restricts the availability of energy cofactors necessary for DMADP formation and isoprene synthesis. Thicker arrows indicate an increased flow of intermediates (black arrows) and energy (red arrows) in response to temperature changes under elevated [CO_2_]. 2‐PG, 2‐phosphoglycolate; G3‐P, glyceraldehyde‐3‐phosphate; Pyr, pyruvate; TP pool, triose phosphate pool.

In this study, we observed that electron flux not directed toward carboxylation exhibits a strong correlation with isoprene emission rates, particularly in *P. tremula* under high thermal stress (40°C) and elevated CO_2_ levels (Figure 4). Under these conditions, excess electrons are increasingly diverted from carbon assimilation to support isoprene synthesis, suggesting a shift in energy allocation under stress. This observation aligns with the model proposed by Rodrigues et al. (2020), which suggests that heat enhances both electron transport and isoprene emissions by channeling surplus reducing power into volatile compound production (overview in Figure 5). The high *Q*_10_ values observed for isoprene emission in *P. tremula* further support this, indicating heightened IspS activity as the temperature rises, providing an outlet for dissipating excess reducing power when photosynthetic carbon fixation becomes constrained.

In *I. edulis* , the relationship between *ETR*‐*J*_v_ and isoprene emission was less pronounced but still significant, suggesting a role for isoprene in managing excess energy under stress. However, the broader thermal tolerance in *I. edulis* leads to more stable photosynthetic responses across CO_2_ gradients, as indicated by overlapping regression points (Figure 4D). This stability likely reflects more robust energy partitioning mechanisms compared to *P. tremula* (de Souza et al. 2024), which exhibited more distinct physiological shifts and clustering of data points under stress (Figure 4C). Notably, the smaller gap between Δ*H*_a_ and *Q*_10_ values for isoprene emission and CO_2_ assimilation in *I. edulis* supports the idea of a more balanced energy budget, consistent with its adaptive response as a tropical species (Dani et al. 2014; Rodrigues et al. 2020; Tiiva et al. 2017; Staudt et al. 2022).

The relatively minor proportion of electrons allocated to isoprene synthesis reinforces the notion that it is not a dominant sink for reducing power (Rasulov et al. 2016). Instead, isoprene emission reflects the overall chloroplast energy status, and the correlation between *ETR*‐*J*_v_ and isoprene emissions likely signifies how cells distribute electrons across metabolic pathways rather than acting as a primary dissipative process (Figure 5; Calfapietra et al. 2013). This indicates that while isoprene production does contribute to excess energy dissipation, it operates in conjunction with other non‐photosynthetic pathways to manage the chloroplast's energy status (Jardine et al. 2014; Dani et al. 2015).

Recent evidence also suggests that high CO_2_ can impair specific enzymatic steps within the MEP pathway, particularly the conversion of HMBDP to DMADP, leading to the accumulation of upstream intermediates and reduced isoprene synthesis (Sahu et al. 2023). Although Sahu et al. (2023) reported that this accumulation occurred independently of photosynthetic performance, other studies have found a link between MEP intermediate buildup and feedback inhibition under high CO_2_ (Li and Sharkey 2013b; Rasulov et al. 2016). These contrasting patterns suggest that the relationship between CO_2_ sensitivity of isoprene emission and photosynthetic performance is species‐ and condition‐dependent, varying across taxa and environmental contexts (Lantz et al. 2019).

Even when CO_2_‐induced suppression of isoprene emissions appears decoupled from photosynthesis, it may still reflect internal shifts in chloroplastic energy allocation. As emphasized by Niinemets et al. (2021), even when PSII and PSI activity remain stable, increased CO_2_ assimilation may elevate the demand for ATP and NADPH, thereby reducing their availability for secondary pathways, such as isoprene synthesis. In this context, the MEP pathway may experience a downregulation of its terminal steps due to reduced energy input, especially considering its high *K*_m_ for ATP and NADPH (Rasulov et al. 2016, 2018). Thus, the suppression of isoprene at short‐term exposure to high CO_2_ may result less from direct enzymatic inhibition or structural limitations in photosynthesis, and more from dynamic energy reallocation within the chloroplast.

Conversely, under high‐temperature stress, *ETR* may continue to rise even after photosynthetic assimilation has plateaued, as shown by Rodrigues et al. (2020) and Sun et al. (2013). This surplus of electrons can generate excess reducing power, which is partially redirected toward isoprene synthesis (Niinemets et al. 1999b; Morfopoulos et al. 2014; Dani et al. 2014; Rasulov et al. 2016). This dynamic is supported by the strong correlation observed between *ETR*‐*J*_v_ and isoprene emissions (Figure 4), indicating that CO_2_‐induced suppression of isoprene emission is modulated by the preferential allocation of ATP and reducing power within the chloroplast (Figure 5).

These observations reinforce the view that isoprene emissions are particularly sensitive to the balance between reducing power availability and enzymatic regulation at key metabolic nodes. For instance, while the DMADP pool tends to decline above the temperature optimum for electron transport, an increase in IspS activity at elevated temperatures—observed both in our data and by Rasulov et al. (2010)—may partially compensate for substrate limitations. This coordination between energetic demand and enzyme kinetics helps explain why CO_2_ suppression of isoprene emissions weakens as temperatures rise. The redistribution of reducing equivalents to non‐carboxylative sinks, such as isoprene synthesis, thus operates in tandem with enhanced IspS activity to mitigate suppression under thermal stress.

Taken together, our findings suggest that differences in CO_2_ sensitivity between species may reflect distinct physiological adaptations in the allocation of energy and regulation of the MEP pathway. By integrating both energetic and enzymatic controls, this study offers mechanistic insights into the modulation of isoprene emissions under combined temperature and CO_2_ concentration, expanding upon the conclusions of Sahu et al. (2023).

### 4.4 Implications for Climate Change and Future Research

These findings contribute to growing evidence that complex physiological processes drive isoprene emissions in response to rising CO_2_ and temperatures. While our results are based on *I. edulis* and *P. tremula* , these species provide contrasting models to explore how thermal tolerance modulates the interplay between CO_2_ suppression and isoprene emissions. Tropical species, such as *I. edulis* , exhibit more stable emissions across environmental gradients, whereas temperate species, like *P. tremula* , show greater sensitivity.

The observed reduction in CO_2_‐induced suppression with rising temperatures appears to follow a threshold pattern, with minimal suppression occurring at temperatures of 35°C or higher, diminishing by approximately 1°C increments beyond this point (Staudt et al. 2022). Our results indicate that the extent of CO_2_ suppression is closely tied to species‐specific thermal tolerance. In less thermally tolerant species, suppression may diminish within a narrower temperature range (e.g., around 30°C–35°C), whereas in more heat‐tolerant species, suppression may persist up to 40°C or beyond. This broader range in CO_2_ suppression likely reflects the ability of heat‐tolerant species to partition energy more effectively toward carbon assimilation under stress, as their thermal tolerance enables greater flexibility in managing reducing power and ATP demands (Dani et al. 2014; Rodrigues et al. 2020; Tiiva et al. 2017). A similar CO_2_‐dependent modulation of isoprene emission was observed in black poplar, where emission rates increased sigmoidally with rising CO_2_ at high temperatures, plateauing beyond 800 μmol mol^−1^, possibly due to enzymatic or substrate limitations (Portillo‐Estrada 2024).

Significantly, we recognize the limitation of drawing broad conclusions from just two species. Rather than generalizing, our study highlights key mechanisms underlying the loss of CO_2_ suppression as temperature rises above species‐specific tolerance thresholds. These insights underscore the need for further research to evaluate whether similar processes occur in other species across diverse ecosystems. For instance, the reliance on surplus reducing power under thermal stress, where photosynthetic carbon reduction is limited, may drive increased isoprene emissions in various contexts. Similar mechanisms operate on a larger scale in the Amazon rainforest during the dry season (Lian et al. 2024). These findings suggest that the principles observed here could extend beyond the studied species but require further validation in broader ecological settings.

While other physiological processes, such as stomatal behavior, may also modulate CO_2_ responses (Pegoraro et al. 2004; Lantz et al. 2019; Staudt et al. 2022; Sahu et al. 2023), our results indicate that the dominant controls under thermal stress are linked to the internal energy distribution and regulation of the MEP pathway. These findings highlight that species‐specific differences in photosynthetic heat tolerance thresholds play a key role in shaping isoprene responses under elevated CO_2_. By identifying core regulatory mechanisms, this work aims to stimulate broader investigations into how temperature and CO_2_ interact to influence isoprene emissions across diverse taxa and ecosystems, advancing our understanding of plant physiological responses to climate change.

## 5 Conclusions

This study has addressed critical gaps in our understanding of how thermal tolerance modulates the interplay between CO_2_ suppression and isoprene emissions under elevated CO_2_ and temperature conditions. By revealing the mechanisms governing these responses, particularly the role of electron partitioning and enzymatic controls, our findings indicate that species‐specific thermal thresholds play a pivotal role in determining the extent of CO_2_ suppression, with *I. edulis* exhibiting broader tolerance ranges and *P. tremula* showing greater sensitivity.

Our results demonstrate that temperatures exceeding the photosynthetic optimum weaken or eliminate the CO_2_ suppression of isoprene emissions through a combination of energetic redistribution and enzymatic regulation. Enhanced electron availability within the chloroplast and increased IspS activity act in tandem to compensate for substrate limitations under thermal stress, reflecting a complex interaction between energy partitioning and enzymatic controls. These findings refine existing hypotheses by highlighting the role of thermal tolerance in modulating these dynamics and expanding our understanding of how energy imbalances drive isoprene emission responses at elevated temperatures.

While this study reinforces current knowledge by identifying key physiological mechanisms, it is based on two contrasting species. Future studies should expand this framework to include a broader range of taxa and environmental conditions, thereby clarifying whether the observed patterns are generalizable across plant systems. Investigating how leaf‐level processes scale to ecosystem dynamics will also be essential for improving predictive models of biogenic volatile organic compound emissions under climate change scenarios. Such efforts will provide critical tools not only for understanding plant responses to combined CO_2_ and temperature stresses but also for providing a framework for future research.

## Conflicts of Interest

The authors declare no conflicts of interest.

## Supporting information


**Figure S1.** Temperature sensitivity net assimilation rate (A, B), electron transport rate (*ETR*) (C, D), and electron flux used for the carboxylation (*J*_v_) (E, F) in *Populus tremula* (aspen) and *Inga edulis* (ice‐cream‐bean, ingá‐cipó) across temperatures at different CO_2_ concentration.
**Figure S2.** Temperature sensitivity of isoprene emissions rate (A, B), DMADP pool size (C, D), and isoprene synthase activity (E, F) in *Populus tremula* and *Inga edulis* across temperatures at different CO_2_ concentrations. Data presentation and statistical significance follow the same conventions described in Figure S1.
**Figure S3.** Relationships between electron transport rate and intercellular CO_2_ concentration (*C*_i_) for *Populus tremula* (A) (European aspen) and *Inga edulis* (B) (ice‐cream‐bean, ingá‐cipó).

## Data Availability

The datasets supporting this article will be made publicly available in an open repository upon publication.
