# Supplementary material for: Photosynthetic Temperature Tolerance Threshold Determines How Isoprene Emission is Affected by Elevated CO2 Concentration at High Temperatures
Source: Plant Environ Interact. 2025 May 2;6(3):e70053. doi: 10.1002/pei3.70053 (PMC12046568; doi:10.1002/pei3.70053)
Supplement: Supplementary file 1 — Figure S1. Temperature sensitivity net assimilation rate (A, B), electron transport rate (ETR) (C, D), and electron flux used for the carboxylation (J v) (E, F) in Populus tremula (aspen) and Inga edulis (ice‐cream‐bean, ingá‐cipó) across temperatures at different CO2 concentration. Figure S2. Temperature sensitivity of isoprene emissions rate (A, B), DMADP pool size (C, D), and isoprene synthase activity (E, F) in Populus tremula and Inga edulis across temperatures at different CO2 concentrations. Data presentation and statistical significance follow the same conventions described in Figure S1. Figure S3. Relationships between electron transport rate and intercellular CO2 concentration (C i) for Populus tremula (A) (European aspen) and Inga edulis (B) (ice‐cream‐bean, ingá‐cipó). [file PEI3-6-e70053-s001.docx]

Supplementary Information for Plant-Environment Interactions

**Article title:** Photosynthetic temperature tolerance threshold determines how isoprene emission is affected by elevated CO_2_ concentration at high temperatures

**Short title:** Isoprene emission under high temperature and CO_2_

**Authors:** Vinícius Fernandes de Souza^1*^, José Francisco de Carvalho Gonçalves^2^, Bakhtier Rasulov^1,^, Eero Talts^1^, Catherine Morfopoulos^4^, Sergio Duvoisin Junior^3^, Patrícia Melchionna Albuquerque^3^, Ülo Niinemets^1,5^

**Affiliations:**

^1^Institute of Agricultural and Environmental Sciences, Estonian University of Life Sciences, Kreutzwaldi 1, Tartu 51006, Estonia. V.F.S., [vinicius.fernandesdesouza@emu.ee](mailto:vinicius.fernandesdesouza@emu.ee); B.R., [bakhtier.rasulov@ut.ee](mailto:bakhtier.rasulov@ut.ee); E.T., [Eero.Talts@emu.ee](mailto:Eero.Talts@emu.ee); Ü.N., [ylo.Niinemets@emu.ee](mailto:ylo.Niinemets@emu.ee).

^2^Laboratory of Plant Physiology and Biochemistry, National Institute for Amazonian Research - INPA, Araújo Avenue, 2236, Aleixo, Manaus, AM 69.011-970, Brazil. J.F.C.G., [jfc@inpa.gov.br](mailto:jfc@inpa.gov.br).

^3^Amazonas State University, Ave. Darcy Vargas, 2100, Manaus, AM 69050-010, Brazil. P.M.A., [patialbuq@hotmail.com](mailto:patialbuq@hotmail.com). S.D.J., [duvoisin66@hotmail.com](https://d.docs.live.net/55796f5dd98ba049/Documents/Estonia/Article%20works/Isoprene%20Populus%20vs%20Inga/Jounal_2024/Version%20submitted_Plant%20Biology/duvoisin66@hotmail.com).

^4^Imperial College of London, Department of Life Sciences (Silwood Park), Berks, United Kingdom of Great Britain – England, Scotland, Wales. C.M., [c.morfopoulos@imperial.ac.uk](file:///C:\Users\vinic\Downloads\c.morfopoulos@imperial.ac.uk).

^5^Estonian Academy of Sciences, Kohtu 6, 10130 Tallinn, Estonia.

**ORCID IDs: 0000-0002-7146-5492 (V.F.S.); 0000-0001-9197-4617 (J.F.C.G.); 0000-0001-5178-8617 (B.R.); 0000-0002-8093-6444 (E.T.); 0000-0002-6121-2483 (C.M.); 0000-0003-2577-7898 (S.D.J.); 0000-0001-8614-7676 (P.M.A.); 0000-0002-3078-2192 (Ü.N.).**

^*^**Author for Contact details**: Vinícius Fernandes de Souza ([vinicius.fernandesdesouza@emu.ee](mailto:vinicius.fernandesdesouza@emu.ee)).

The author responsible for the distribution of materials integral to the findings presented in this article following the policy described in the Instructions for Authors is Vinícius Fernandes de Souza.

**This PDF file includes:**

Figs. S1 to S3.

**Supplementary Figures**


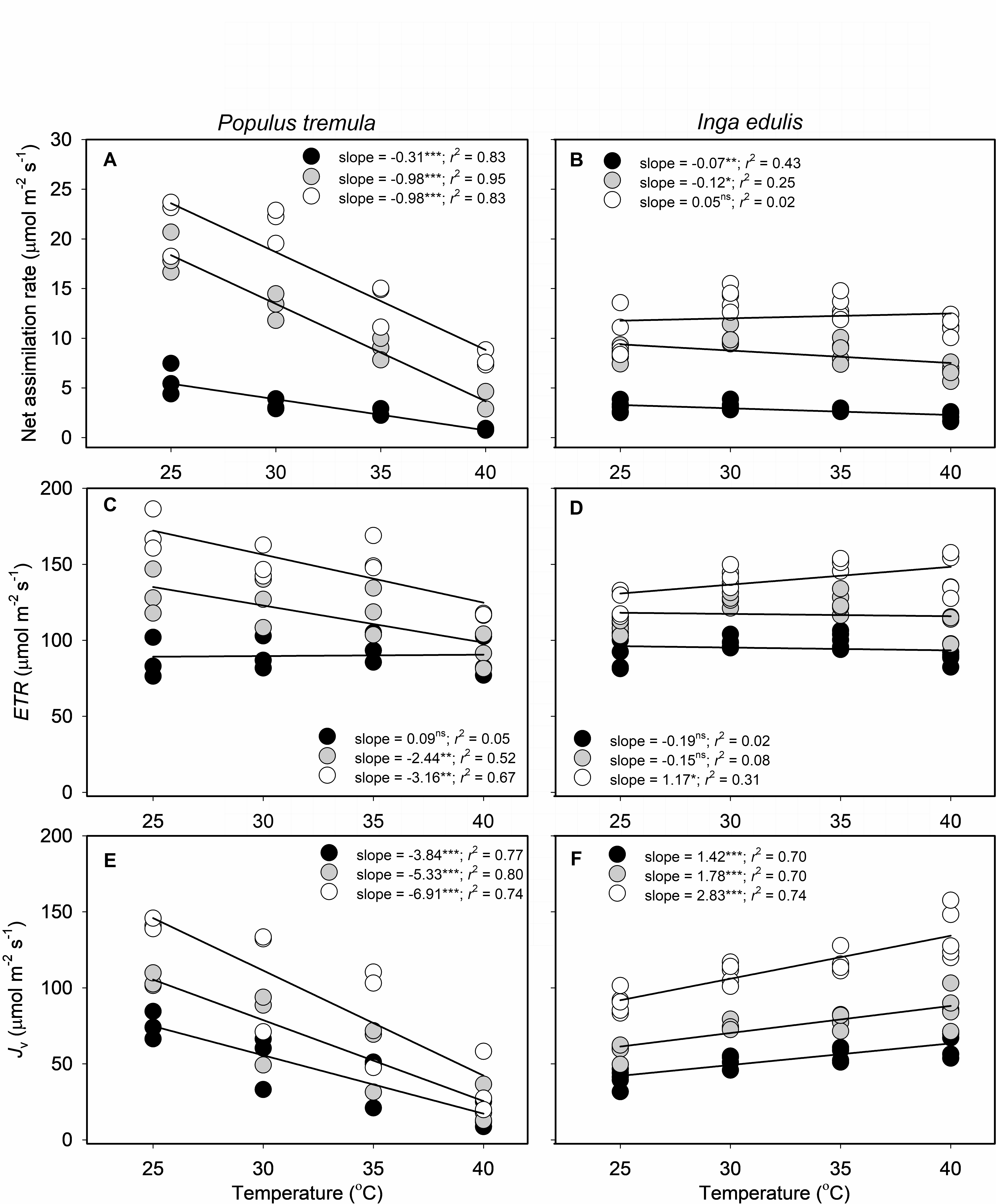


**Supplementary Fig. S1**. Temperature sensitivity net assimilation rate (A, B), electron transport rate (*ETR*) (C, D), and electron flux used for the carboxylation (*J*_v_) (E, F) in *Populus tremula* (aspen) and *Inga edulis* (ice-cream-bean, ingá-cipó) under three CO_2_ concentrations: 150 µmol mol^-1^ (black), 400 µmol mol^-1^ (grey), and 1000 µmol mol^-1^ (white). Panels on the left (A, C, E) correspond to *Populus tremula* and those on the right (B, D, F) to *Inga edulis*. Regression lines show the relationship at CO_2_ concentrations of 150 (black), 400 (grey), and 1000 µmol mol^-1^ (white) across four temperatures (25 °C, 30 °C, 35 °C, and 40 °C). The slopes and *r*² values for each regression are reported for each CO_2_ treatment. Asterisks indicate levels of statistical significance (**P* < 0.05; ***P* < 0.01; ****P* < 0.001).





**Supplementary Fig. S2**. Temperature sensitivity of isoprene emissions rate (A, B), DMADP pool size (C, D), and isoprene synthase activity (E, F) in *Populus tremula* and *Inga edulis* under three CO_2_ concentrations: 150 µmol mol^-1^ (black), 400 µmol mol^-1^ (grey), and 1000 µmol mol^-1^ (white). Panels on the left (A, C, E) correspond to *Populus tremula* and those on the right (B, D, F) to *Inga edulis*. Data presentation and statistical significance follow the same conventions described in Supplementary Figure S1.


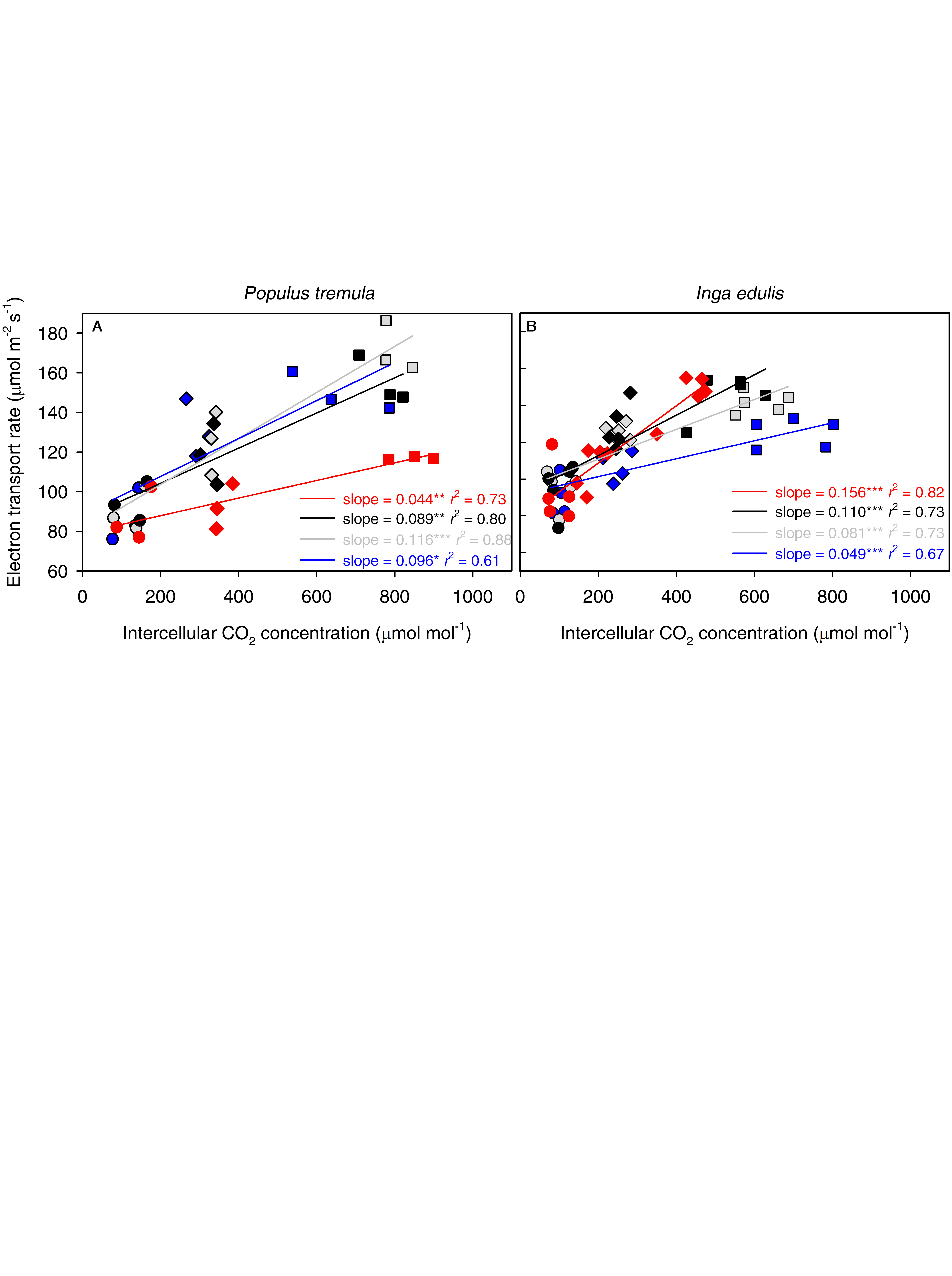


**Supplementary Fig. S3.** Relationships between electron transport rate and intercellular CO_2_ concentration (*C*_i_) for *Populus tremula* (A) (European aspen) and *Inga edulis* (B) (ice-cream-bean, ingá-cipó). The data were fitted by linear regression for each temperature value: 25 ºC (blue), 30 ºC (gray), 35 ºC (black), 40 ºC (red). Different symbols correspond to the three atmospheric CO_2_ concentrations (circle, 150 µmol mol^-1^; diamond, 400 µmol mol^-1^; and square, 1000 µmol mol^-1^). Coefficients of determination and significance of slopes are given. Asterisks indicate levels of statistical significance (**P* < 0.05; ***P* < 0.01; ****P* < 0.001), and 'ns' denotes non-significant results.
